# Multi-omics analysis explores the potential mechanisms of *Huangtcia renifolia* ethanolic extract against osteoporosis in ovariectomized rats

**DOI:** 10.3389/fendo.2026.1795581

**Published:** 2026-09-30

**Authors:** Liping Yang, Tian Xiao, Zhuo Zhang, Xiaohua Duan

**Affiliations:** Yunnan Key Laboratory of Dai and Yi Medicines, Yunnan University of Chinese Medicine, Kunming, Yunnan, China

**Keywords:** bone-muscle crosstalk, gut microbiota, *Huangtcia renifolia*, metabolomics, osteoporosis, transcriptomics

## Abstract

**Background:**

*Huangtcia renifolia* (L.) H. Ohashi & K. Ohashi, a traditional folk medicine, has been used for centuries by the Dai people in Xishuangbanna, Yunnan Province, China. Traditionally, it has been used to clear kidney heat, promote diuresis, and treat conditions, including kidney deficiency, sexual dysfunction, hepatitis, and osteoporosis. Recent pharmacological investigations indicate that *Huangtcia renifolia* ethanol extract (DRS) exerts bone-protective effects, partially via modulation of OPG/RANKL/RANK and BMP-2/Smad signaling pathways. This study used an integrated multi-omics strategy to explore the bone-protective effects of DRS and its potential mechanisms in ovariectomized (OVX) rats.

**Methods:**

The chemical composition of DRS was profiled by widely targeted UPLC-MS/MS. Osteoporosis was induced in rats via bilateral ovariectomy, and bone status was assessed using serum biochemical analyses, micro-computed tomography (microCT) and hematoxylin-eosin (H&E) staining of femur tissue. Integrated multi-omics analyses were subsequently performed, comprising 16S rRNA sequencing of fecal microbiota, widely targeted serum metabolomics, and femoral transcriptomic sequencing. *In vitro* validation was performed using MC3T3-E1 osteoblasts treated with DRS-containing serum.

**Results:**

DRS treatment significantly increased bone mineral density, improved trabecular bone microstructure, and effectively reversed abnormal bone turnover markers in OVX rats. Gut microbiota profiling indicated that DRS treatment modulated the gut microbiota, with significant increases in the relative abundance of *Lactobacillus* and *Limosilactobacillus*. Serum metabolomics identified 34 metabolites differentially abundant in DRS-treated rats, with pathway enrichment analyses highlighting purine metabolism as one of the principal affected pathways. Femoral transcriptomics and RT-qPCR validation indicated that DRS treatment was associated with upregulation of osteogenic genes (*Col1a1* and *Col1a2*) and downregulation of muscle-related genes (*Myog*, *Myh3*, and *Mstn*), consistent with potential modulation of pathways linking bone and muscle function. Additionally, DRS-containing serum dose-dependently enhanced osteoblast differentiation and upregulated BMP2 and RUNX2 expression *in vitro*.

**Conclusion:**

DRS treatment protects against osteoporosis in OVX rats. This protection was associated with gut microbiota composition, serum metabolic profiles, and the expression balance between osteogenic and muscle-related genes in bone.

## Graphical Abstract

**Figure:**
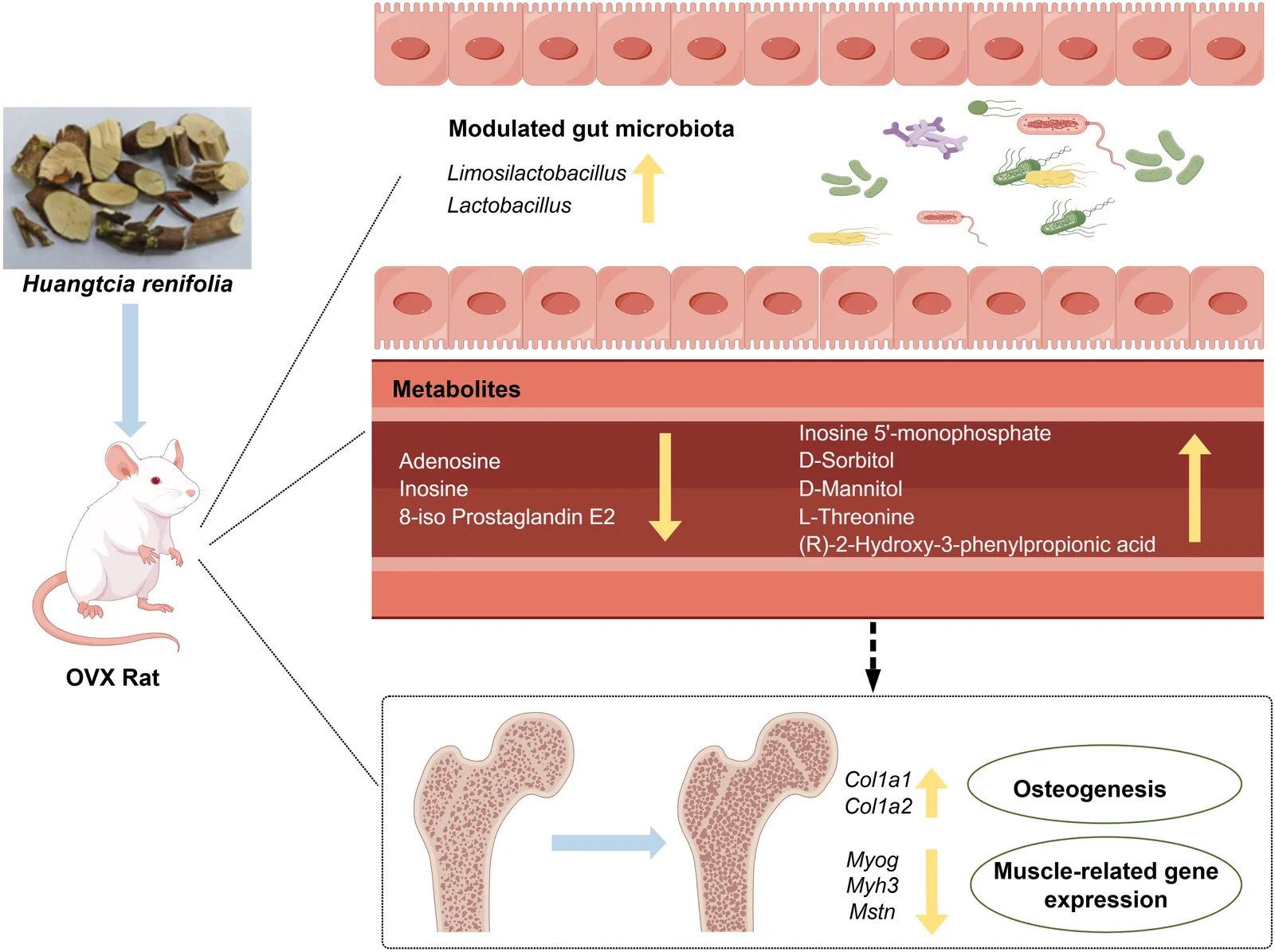

## 1 Introduction

Osteoporosis is a common metabolic bone disease characterized by reduced bone density and deterioration of bone tissue microstructure (1). It frequently coexists with muscle atrophy and physical dysfunction, forming a syndrome known as osteosarcopenia, which markedly elevates fracture risk, disability, and mortality (2). As the global population ages, the prevalence of osteoporosis is increasing. In China, 20.6% of women over the age of 40 suffer from osteoporosis, with postmenopausal females constituting the high-risk population (3, 4). Present treatment methods include estrogen replacement therapy, parathyroid hormone analogues, and bisphosphonates (5). These treatments have demonstrated efficacy in improving bone mineral density (BMD) and reducing fracture risk. However, long-term administration is associated with significant adverse effects, including atypical femoral fractures, hypercalcemia, and increased risks of breast cancer and cardiovascular events (6). Therefore, it is urgent need to investigate and formulate effective and safe drugs for treating osteoporosis.

Osteoporosis pathogenesis is complex and multifactorial, involving complex interactions among genetic factors, hormonal changes, and lifestyle factors (7). A recent study has identified a close bidirectional regulatory network between the gut microbiota and the skeletal system, referred to as the gut-bone axis, which maintains continuous dialogue and mutual regulation through immune, metabolic, and endocrine pathways (8). Gut microbes and their metabolites serve as critical messengers that modulate bone homeostasis (9). When gut homeostasis is disrupted, bone health is adversely affected through multiple pathways: dysbiosis triggers immune dysregulation and RANKL upregulation (10); microbial metabolites directly influence osteoblasts and their function (11); chronic inflammation disrupts the bone formation-resorption equilibrium (12). Conversely, bone-derived signals can modulate intestinal function, and aberrant bone metabolism can induce intestinal stress responses, establishing a vicious cycle that accelerates osteoporotic progression (13).

*Huangtcia renifolia* (L.) H. Ohashi & K. Ohashi, formerly known as *Desmodium renifolium* (L.) Schindl., is a leguminous plant whose dried roots have been traditionally used in Dai medicine. This species is indigenous to southern China, particularly the provinces of Hainan, Yunnan, and Taiwan, and its distribution extends throughout Southeast Asia. In the Dai ethnic region of Xishuangbanna, Yunnan Province, *Huangtcia renifolia* has been traditionally recognized as an effective remedy for alleviating kidney heat, promoting diuresis, reducing jaundice, and strengthening vital energy (14). Dai medicine practitioners frequently use it to treat conditions such as nephritis, uremia, and hepatitis (15). Phytochemical investigation has revealed that *Huangtcia renifolia* is rich in isopentenyl chalcones, including Oxyphyllumchalcones A-C and Renifolins B-I, along with other flavonoids, including licochalcone A and isobavachalcone (16). Additionally, its close relative *Podocarpium podocarpum* has exhibited anti-osteoporotic properties in preclinical studies, demonstrating efficacy in preventing bone loss in ovariectomized (OVX) models and promoting osteoblast proliferation (17). Our previous studies have indicated that *Huangtcia renifolia* ethanol extract (DRS) mitigates osteoporotic pathology in OVX rats by modulating OPG/RANKL/RANK and BMP-2/Smad signaling pathways, an effect further supported by *in vitro* findings of suppressed RANKL-induced osteoclastogenesis in RAW264.7 cells (18, 19). Furthermore, recent studies using *Caenorhabditis elegans* models have revealed that DRS attenuates age-related muscle degeneration and preserves motor function (20). These findings suggest that DRS may exert bone-protective effects through the muscle-bone crosstalk axis, in addition to its direct osteogenic effects.

The present study aimed to explore the potential mechanisms associated with the osteoprotective effects of DRS in OVX rats, using an integrated multi-omics strategy including 16S rRNA gene sequencing, widely targeted serum metabolomics, and femoral transcriptomics. Through this comprehensive approach, we sought to characterize DRS-induced alterations in gut microbial composition, metabolic profiles, and femoral gene expression to better understand the multi-target mechanisms underlying its bone-protective effects.

## 2 Materials and methods

### 2.1 Animals

The animal experiments in this study were approved by the Animal Protection and Use Committee of Yunnan University of Chinese Medicine and conducted in accordance with the current animal welfare guidelines (R-062022221080). Female Sprague-Dawley rats (8 weeks old, 180-200 g) were obtained from Kunming Medical University, China. All rats were housed individually at 22 ± 2 °C, 45%-60% relative humidity, with a 12-h light/dark cycle.

### 2.2 Preparation of DRS

The dried roots of *Huangtcia renifolia* were obtained from the Institute of Ethnic Medicine in Xishuangbanna Dai Autonomous Prefecture, Yunnan Province, China. Botanical authentication was performed by Yin Zili, senior laboratory technician at Yunnan University of Chinese Medicine. A voucher specimen has been deposited in the Yunnan Key Laboratory of Dai and Yi Medicines (SY20250314). The powdered material was extracted with 95% ethanol (1:8, w/v) under reflux for 45 min, and the extraction was repeated three times. Ethanol was removed under reduced pressure, resulting in the DRS extract with an extraction yield of 7% (w/w). The extract was stored at 4°C until subsequent use.

### 2.3 UPLC-MS/MS-based metabolomic analysis of DRS

DRS was thawed at room temperature. Following vortexing, 600 μL of precooled 70% methanol-water with internal standard was added to 50 mg of sample maintained at -20°C. After vortexing (15 min) and centrifugation (12,000 rpm, 4°C, 3 min), the supernatant was filtered (0.22 μm) for LC-MS/MS analysis. Widely targeted metabolomics was performed using UPLC-MS/MS (ExionLC AD, Sciex; QTRAP 6500+) on an Agilent SB-C18 column (1.8 μm, 2.1 × 100 mm) with water and acetonitrile (both with 0.1% formic acid) as mobile phases. The gradient was as follows: 0-1 min, 5% B; 1-10 min, 5%-95% B; 10-11 min, 95% B; 11-12 min, 95%-5% B; 12-14 min, 5% B. Mass detection used electrospray ionization (ESI) in positive/negative modes with multiple reaction monitoring (MRM) transitions. Metabolites were identified against MetWare database (Metware, China).

### 2.4 Establishment of animal model and administration

Female Sprague-Dawley rats underwent bilateral ovariectomy to induce postmenopausal osteoporosis as previously described (21, 22). Under isoflurane anesthesia, 24 rats received bilateral ovariectomy, while eight rats underwent sham surgery consisting of excision of adipose tissue adjacent to the ovaries without ovarian removal. After a recovery period of two weeks, the 24 OVX rats were divided randomly into three groups (n = 8 per group): OVX group, DRS low-dose group, and DRS high-dose group. The eight sham-operated rats served as the Sham group. Sham and OVX control groups were orally administered with 0.5% sodium carboxymethyl cellulose (CMC-Na) solution as a vehicle. In comparison, the DRS groups were administered with DRS suspended in 0.5% CMC-Na at doses of 2.7 and 5.4 g/kg, respectively. Based on body surface area conversion (factor 6.2), the theoretical equivalent dose in rats was 1.03-1.55 g/kg. In our previous study, three doses of DRS, including the equivalent dose (1.35 g/kg), were tested in OVX rats and produced significant improvements in some bone parameters but not others (19). We therefore selected 2× and 4× the equivalent dose (2.7 and 5.4 g/kg) in the present study to establish a clear dose-response relationship. Both doses were well tolerated throughout the 14-week administration, with no mortality or obvious abnormalities in general condition observed.

Following the treatment period, rats were deeply anesthetized through intraperitoneal injection of sodium pentobarbital (40 mg/kg body weight). Under anesthesia, blood samples were collected from the abdominal aorta, bilateral femora were excised, and fecal samples were harvested. All samples were immediately processed and stored at -80 °C for subsequent analysis. Specifically, femoral samples for transcriptomic analysis were carefully dissected to remove all visible skeletal muscle and periosteum, and subjected to gross and microscopic examination to confirm absence of muscle tissue remnants, thereby minimizing skeletal muscle contamination.

The allocation of samples for downstream analyses was as follows. Serum biochemical analyses were performed in all animals (n = 8 per group). Omics analyses were restricted to the Sham, OVX, and high-dose DRS groups, and a subset of animals from each group was randomly selected for each platform, resulting in n = 6 per group for 16S rRNA sequencing, n = 5 for transcriptomics, and for metabolomics, n = 6 for the Sham group and n = 4 for the OVX and DRS groups. For micro-CT and histological analyses, three femora per group were randomly selected. Sample sizes for each analysis are indicated in the corresponding figure legends.

### 2.5 Biochemical detection

Serum levels of estrogen (E2), bone-specific alkaline phosphatase (ALP), osteocalcin (BGP), procollagen type I N-terminal propeptide (PINP), and C-telopeptide of type I collagen (CTX-1) were quantified using commercial assay kits according to the manufacturer’s protocols (Nanjing Jiancheng Bioengineering Institute, Nanjing, China). LPS levels in serum were determined using a LAL endotoxin assay kit (Beyotime, Shanghai, China).

### 2.6 Micro-computed tomography analysis

Three-dimensional microstructural analysis of the distal femur was performed using a micro-computed tomography system (Skyscan 1174, Bruker, Belgium) with the following parameters: source voltage 50 kV, current 800 μA, and resolution 9 μm per pixel. Image reconstruction and three-dimensional analysis were performed using the NRecon and CTAn software packages (Bruker, Belgium). The following trabecular bone parameters were quantified: BMD, bone volume fraction (BV/TV), trabecular number (Tb.N), trabecular thickness (Tb.Th), trabecular separation (Tb.Sp), and structural model index (SMI). All analyses were performed by an investigator blinded to the experimental group allocation.

### 2.7 Histological analysis

Femora were preserved in 10% neutral formalin, decalcified using 10% formic acid (48 h), dehydrated with increasing concentrations of ethanol, embedded in paraffin, and sliced into 4 μm sections. These sections were then stained with hematoxylin-eosin (H&E) and examined under a light microscope.

### 2.8 High-throughput sequencing for 16S rDNA

Total DNA was extracted from fecal samples using the CTAB/SDS method. The purity of the DNA was assessed through agarose gel electrophoresis, and samples were diluted to a concentration of 1 ng/μL. PCR amplification of the V3-V4 region was performed using barcoded primers and Phusion^®^ High-Fidelity PCR Master Mix. The PCR products were confirmed using 2% agarose gel electrophoresis; qualified products were purified with magnetic beads, quantified, and pooled in equimolar ratios. Libraries were constructed using the TruSeq^®^ DNA PCR-Free Sample Preparation Kit and sequenced on an Illumina NovaSeq 6000 platform. Raw data were filtered to obtain effective sequences, and the sequences were then denoised to generate amplicon sequence variants (ASVs). Taxonomic annotation was performed using the SILVA138 database through the Mothur method. Sequence data were analyzed using QIIME2 and R software (version 4.3.2). Between-group differences in β-diversity were assessed by PERMANOVA with 999 permutations on Bray-Curtis and weighted UniFrac distance matrices, and P values from pairwise comparisons were adjusted using the Benjamini-Hochberg method. Differential taxa were identified by LEfSe (nominal P < 0.05, LDA score > 4) as an exploratory biomarker analysis, and P values from taxonomic comparisons were adjusted using the Benjamini-Hochberg method.

### 2.9 Serum metabolomics

Serum samples (50 μL) were mixed with 300 μL ice-cold 20% methanol-acetonitrile (v/v) containing internal standards, vortexed, and centrifuged (12,000×g, 4°C, 10 min). Supernatants were collected, frozen at -20°C for 30 min, re-centrifuged (12,000×g, 3 min), and analyzed by LC-MS/MS (ExionLC AD coupled to QTRAP 6500+, Sciex). The chromatographic separation utilized Waters HSS T3 (reversed-phase) and BEH Amide (HILIC) columns with gradient elution. Mass detection was performed using ESI in positive and negative modes with MRM transitions. Data were processed with MultiQuant software. Multivariate analysis was performed using R software. Differential metabolites (VIP > 1, fold change ≥ 2 or ≤ 0.5) were subjected to Kyoto encyclopedia of genes and genomes (KEGG) pathway analysis.

### 2.10 Transcriptome sequencing

Total RNA was extracted using Trizol reagent, and RNA quality was determined. Strand-specific RNA-seq libraries were constructed using the KAPA Stranded RNA-Seq Library Prep Kit with the dUTP method. Library quality was evaluated using an Agilent 2100 Bioanalyzer, and quantity was determined by qPCR. Libraries passing quality control (QC) were pooled and sequenced (paired-end, 2×150 bp) on an Illumina HiSeq platform. Raw reads were filtered using fastp (version 0.23.2) and aligned to the Rattus norvegicus reference genome (mRatBN7.2, Ensembl release 109) using HISAT2 (version 2.2.1). Gene-level raw read counts were generated using featureCounts (version 2.0.3) and used as the input for differential expression analysis. Differential expression analysis was performed using DESeq2 (version 1.38.3) with five biological replicates per group and P values were adjusted for multiple testing using the Benjamini-Hochberg method. For stringent differential expression analysis, genes with |log2 fold change| ≥ 1 and adjusted P value (padj) < 0.05 were considered statistically significant. GSEA was performed using clusterProfiler (version 4.6.0) on all genes ranked by log2 fold changes, and gene sets with adjusted P < 0.05 were considered significantly enriched. Genes with |log2 fold change| ≥ 1 and nominal P < 0.05 were used for exploratory GO and KEGG overrepresentation analyses, for which q values were used to assess enrichment significance. FPKM values were used only for expression visualization and correlation analyses, not as the input for DESeq2.

### 2.11 Real-time quantitative PCR

Total RNA was extracted from rat femoral bone tissue using a commercially available RNA extraction kit. After determining RNA concentration and purity, 1 μg of RNA was reverse-transcribed into cDNA using the 2× RTOR-Easy™ Mix. Amplification reactions were performed with Fast SYBR Green qPCR Master Mix UDG on a real-time PCR system. GAPDH served as the reference gene for normalization purposes. The relative expression levels of genes were determined using the 2^−ΔΔCt^ method, with primer sequences provided in **Table 1**.

**Table 1:** Primers.

Gene	Primer sequences
*Col1a1*	Forward: ACCAGCATCACCCTTAGCAC
	Reverse: CTGGTCCTGATGGCAAAACT
*Myog*	Forward: CCAACCCAGGAGATCATCGC
	Reverse: GCTTGCAGCTGTACTGGTTG
*Myh3*	Forward: AGAGCCTGAGGAAGAGCGAG
	Reverse: GGTGTCGTAGTTGTCCAGCA
*Mstn*	Forward: GACCTCCTACAACAGCAACCAG
	Reverse: CATCAAACTCCTGGATGGCG
*GAPDH*	Forward: AGGTCGGTGTGAACGGATTTG
	Reverse: GGGGGTCGTTGATGGCAACA

### 2.12 Cell culture experiments

Drug-containing serum was prepared as described (18). Serum was collected from DRS-treated rats (2.7 and 5.4 g/kg) or vehicle-treated rats 2 hours after the final administration via abdominal aorta puncture under deep anesthesia. Blood was allowed to clot at room temperature for 30 min, then centrifuged at 3,000×g for 15 min. The supernatant serum was harvested, heat-inactivated at 56 °C for 30 min, and stored at -80 °C until use.

MC3T3-E1 cells (Cell Bank of the Chinese Academy of Sciences) were cultured in α-MEM with 10% FBS and 1% penicillin-streptomycin at 37 °C, 5% CO_2_. Cells were seeded at 5 × 10^4^ cells/well in 6-well plates and grown to confluence, then switched to osteogenic medium (containing β-glycerophosphate, ascorbic acid, and dexamethasone) supplemented with 10% vehicle serum (control) or drug-containing serum. The basal group was maintained in α-MEM with 10% FBS. Cells were cultured for 7 days prior to analysis, with medium replacement every 3 days. After 7 days, ALP staining was performed. For Western blot, total protein was extracted, separated by SDS–PAGE, transferred to PVDF, and probed for BMP2 and RUNX2 (primary antibodies 1:1000, Cell Signaling Technology) with ECL detection.

### 2.13 Statistical analysis

Non-omics data were analyzed using GraphPad Prism 8.0 and are presented as mean ± standard error of the mean (SEM). Comparisons among multiple groups were performed using one-way analysis of variance (ANOVA) followed by Tukey’s *post-hoc* test. For non-omics data, P < 0.05 was considered statistically significant.

For correlation analyses, Spearman’s rank correlation was used for associations between gut microbiota, serum metabolites and serum biochemical indices, and Pearson’s correlation was used for associations between transcriptomic and metabolomic data. Individually matched samples from the same animals were used for cross-platform correlation analyses (n = 4 per group for microbiome–metabolome and transcriptome–metabolome correlations; n = 3 per group for correlations involving serum biochemical indices). Given the limited number of paired samples, these analyses were considered exploratory; nominal P values (<0.05) were used without adjustment for multiple comparisons, and the results should be interpreted with caution. All correlation analyses were performed using the online Metware Cloud platform (https://cloud.metware.cn/).

## 3 Results

### 3.1 Analysis of potential constituents of DRS by UPLC-MS/MS

DRS was subjected to widely targeted metabolomics analysis using UPLC-MS/MS coupled to a QTRAP mass spectrometer in MRM mode, revealing 1,575 metabolites. These metabolites were classified as follows: 308 flavonoids, 219 phenolic acids, 201 lipids, 145 alkaloids, 131 amino acids and derivatives, 95 lignans and coumarins, 94 terpenoids, 76 organic acids, 55 nucleotides and derivatives, 35 quinones, 5 tannins, 4 steroids, and 207 other substances. The major metabolites in DRS, as well as the total ion chromatograms (TICs) analyzed by positive and negative ion mass spectrometry, are presented in **Figure 1**; **Table 2**. The metabolites with the highest relative abundance in DRS included N-(1-deoxy-1-fructosyl) leucine and crotalarin, as determined by peak area comparison.

**Figure 1:**
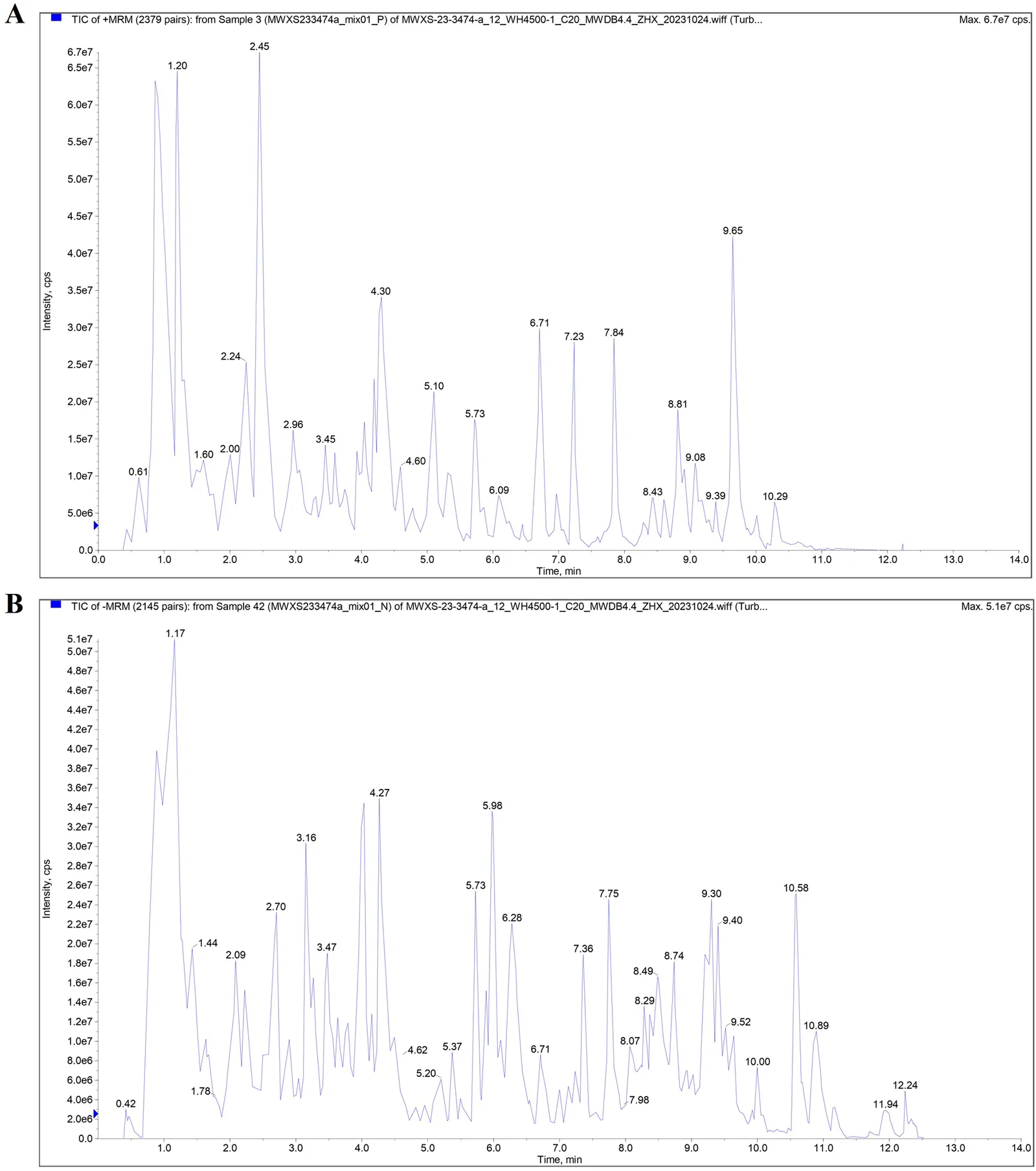
TIC plots of mixed QC samples of DRS. **(A)** Positive ion mode. **(B)** Negative ion mode. The horizontal coordinate is the retention time (RT) of the metabolite assay, and the vertical coordinate is the intensity of the ion current of the ion assay (intensity in cps, counts per second).

**Table 2:** The top 20 metabolites of DRS.

Compounds	Formula	Class I	Class II	Relative content
N-(1-Deoxy-1-fructosyl)Leucine	C_12_H_23_NO_7_	Amino acids and derivatives	Amino acids and derivatives	697795795.3
Crotalarin	C_20_H_18_O_5_	Flavonoids	Isoflavones	313842313.9
4’-Hydroxy-5,7-dimethoxyflavanone	C_17_H_16_O_5_	Flavonoids	Flavanones	308070606.6
2-Hydroxy-4-methyl-3-undecanoyloxypentanoic acid methyl ester	C_18_H_34_O_5_	Lipids	Free fatty acids	306402656.8
9,12,13-Trihydroxy-10-octadecenoic acid	C_18_H_34_O_5_	Organic acids	Organic acids	296723520.2
Vicenin-3	C_26_H_28_O_14_	Flavonoids	Flavones	282254837.3
Pyran[3,4-b]indole-2-ketone	C_11_H_9_O_2_N	Alkaloids	Plumerane	279037649.3
N-(1-Deoxy-1-fructosyl)Phenylalanine	C_15_H_21_NO_7_	Amino acids and derivatives	Amino acids and derivatives	278148289.4
p-Coumaroylagmatine	C_14_H_20_N_4_O_2_	Alkaloids	Phenolamine	277205639.2
3,5-Dihydro-2H-Furo[3,2-C]Quinolin-4-One	C_11_H_9_NO_2_	Alkaloids	Quinoline alkaloids	265809328.9
Machilusolide D	C_18_H_30_O_3_	Others	Lactones	259310286.9
2,5-Dihydroxybenzoic acid-5-O-glucoside	C_13_H_16_O_9_	Phenolic acids	Phenolic acids	256458008.8
Chrysin-5-O-glucoside (Toringin)	C_21_H_20_O_9_	Flavonoids	Flavones	255558822.8
Protocatechuic acid-4-O-glucoside	C_13_H_16_O_9_	Phenolic acids	Phenolic acids	254056582
3-amino-2-naphthoic acid	C_11_H_9_NO_2_	Alkaloids	Alkaloids	246832732
3-Indoleacrylic acid	C_11_H_9_NO_2_	Alkaloids	Plumerane	246758758.2
Methylillicinone F	C_16_H_22_O_4_	Lignans and Coumarins	Coumarins	229922687.3
9,10,13-Trihydroxy-11-Octadecenoic Acid	C_18_H_34_O_5_	Lipids	Free fatty acids	229781151
5S,8R-DiHODE; (5S,8R,9Z,12Z)-5,8-Dihydroxyoctadeca-9,12-dienoate	C_18_H_32_O_4_	Lipids	Free fatty acids	220865346.4
Hydroperoxylinoleic acid	C_18_H_32_O_4_	Lipids	Free fatty acids	220854955.1

### 3.2 DRS modulates serum biochemical parameters in OVX rats

Ovariectomy led to a notable decrease in serum E2 levels, which were restored by DRS treatment (**Figure 2**). Compared to the Sham group, OVX rats exhibited markedly elevated BGP and CTX-1 levels, along with significantly reduced PINP levels. DRS effectively reversed these alterations. In addition, serum LPS levels were significantly elevated in OVX rats and reduced by DRS treatment.

**Figure 2:**
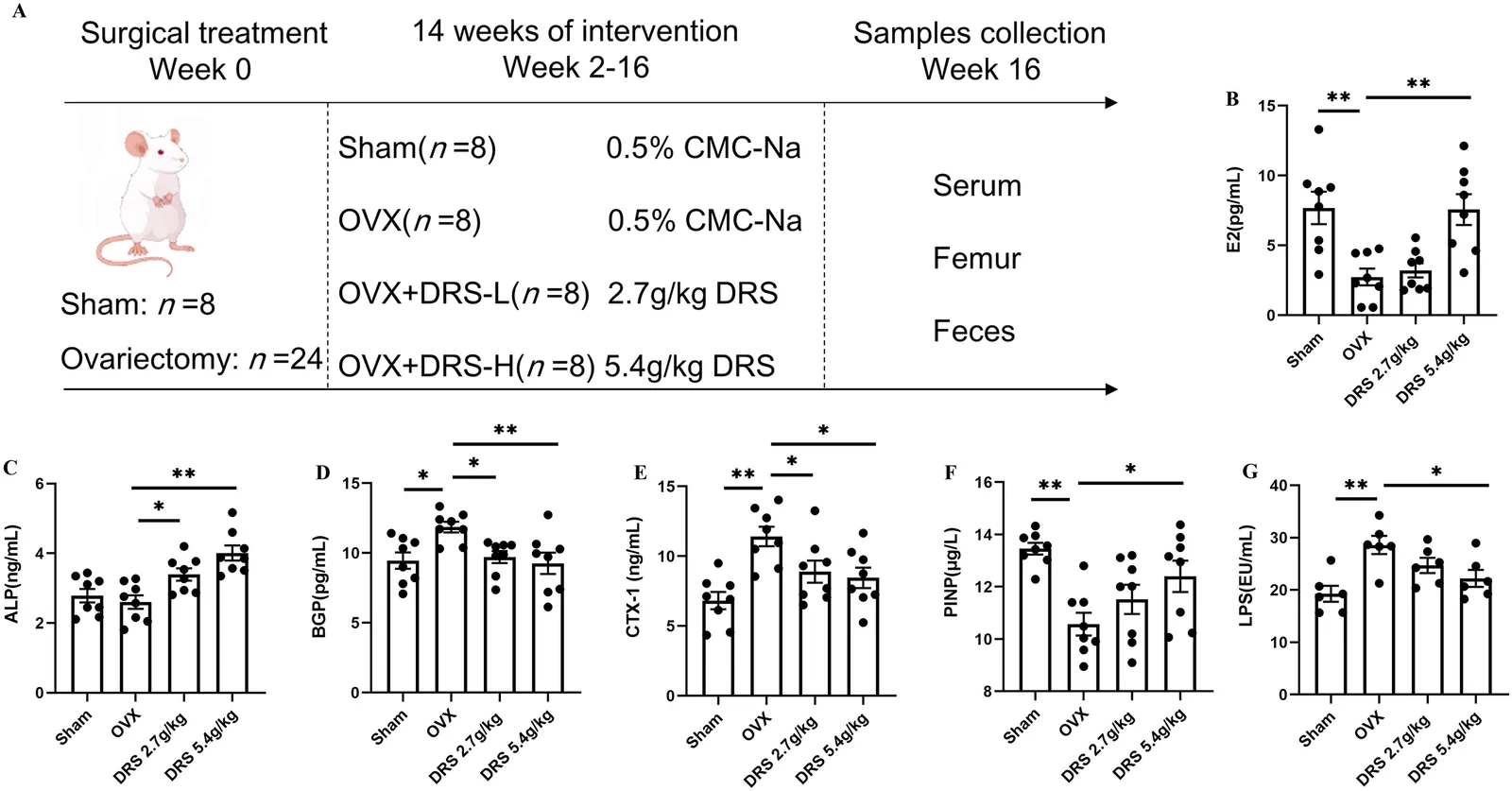
Effects of DRS on serum parameters in OVX rats. **(A)** Schematic diagram of experimental design. Serum **(B)** E2, **(C)** ALP, **(D)** BGP, **(E)** CTX-1, **(F)** PINP, and **(G)** LPS levels. Data are presented as mean ± SEM (n = 8). ^*^P < 0.05, ^**^P < 0.01. The rats in the schematic diagram of the experimental design were created with Figdraw.

### 3.3 DRS improved bone microstructure in OVX rats

To further investigate bone microstructure, we used micro-CT to evaluate femoral bone architecture in rats. The Sham group exhibited tightly arranged, well-structured trabeculae with uniform thickness and orientation (**Figure 3A**). Following ovariectomy, rats displayed significantly reduced trabecular number, increased trabecular fragmentation, disordered arrangement, and evident cavities. DRS treatment significantly ameliorated this structural deterioration. Analysis of micro-CT parameters (**Figures 3B–G**) revealed that the OVX group had significantly lower BMD, BV/TV, and Tb.N, but higher Tb.Sp and SMI compared to the Sham group. Conversely, the high-dose DRS group significantly reversed these changes. Notably, Tb.Th exhibited no alterations across all groups. H&E staining of the distal femur (**Figures 3H, I**) confirmed these findings. The Sham group displayed tightly arranged and structurally intact trabeculae. In contrast, the OVX group exhibited reduced trabecular number, sparse distribution, and evident fragmentation. Furthermore, following 14 weeks of DRS treatment, rats exhibited significantly increased trabecular density and connectivity, particularly in the high-dose group. These findings collectively demonstrate that DRS significantly mitigates ovariectomy-induced osteoporosis.

**Figure 3:**
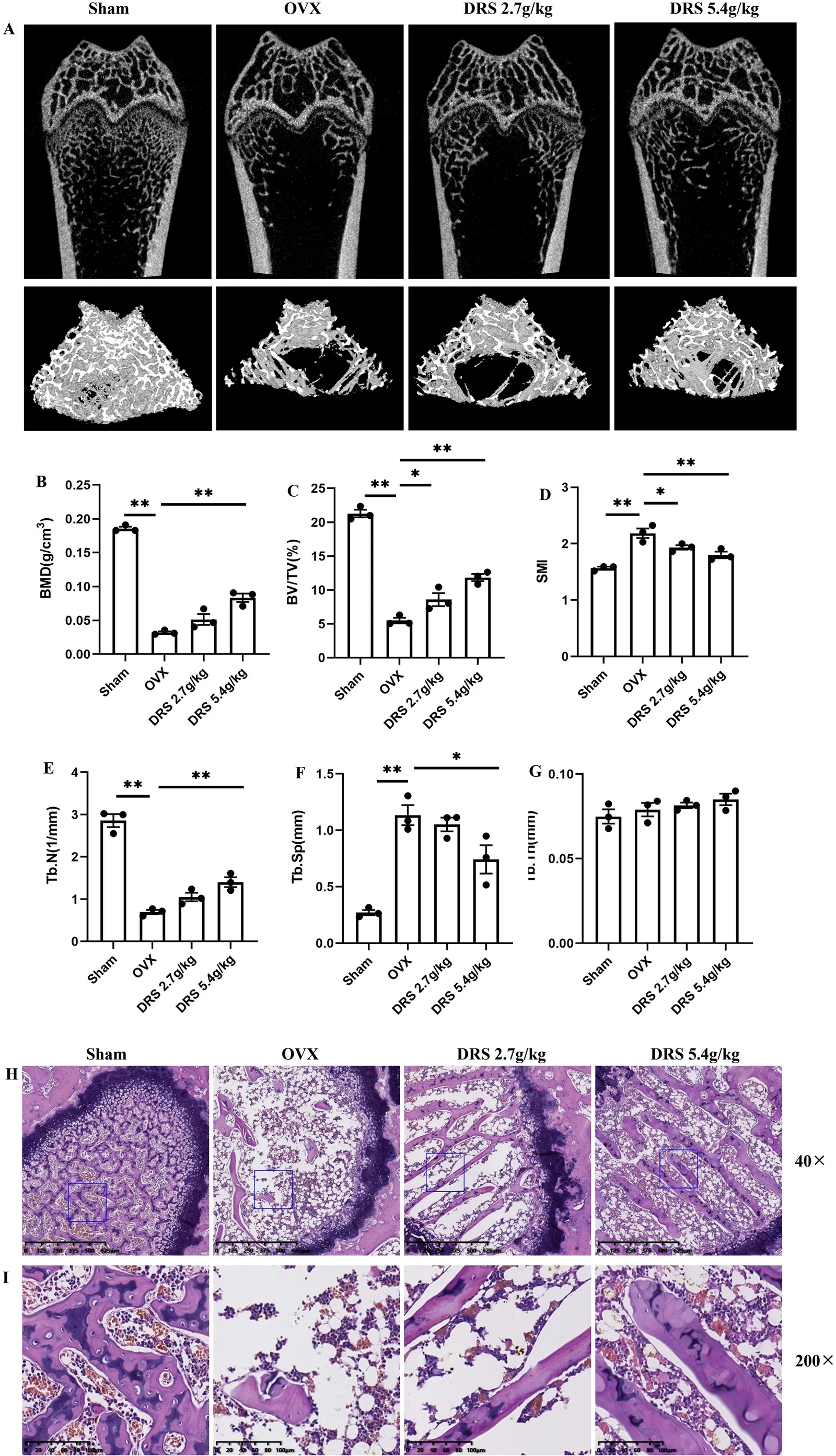
DRS improves bone microstructure in OVX rats. **(A)** Representative micro-CT images of rat femurs. Quantitative analysis of **(B)** Trabecular BMD in femur, **(C)** BV/TV, **(D)** SMI, **(E)** Tb.N, **(F)** Tb.Sp, **(G)** Tb.Th, **(H)** 40×, and **(I)** 200× magnification of femoral H&E staining histopathological images. Data are presented as mean ± SEM (n = 3). *P < 0.05, **P < 0.01.

### 3.4 DRS alters the composition of gut microbiota in OVX rats

To investigate the effects of DRS on the gut microbiota in OVX rats, we selected 18 fecal samples from Sham, OVX, and high-dose DRS groups for 16S rDNA sequencing analysis (n = 6 per group). The Venn diagram (**Figure 4A**) revealed 865 shared ASVs across all three groups, with 28, 43, and 31 unique ASVs identified in Sham, OVX, and DRS groups, respectively. Furthermore, α-diversity analysis showed numerical variations among groups; however, no statistically significant differences were observed (**Supplementary Figures 1B−G**). Principal coordinates analysis (PCoA) illustrated both similarities and distinctions in gut microbial communities across the three groups (**Figures 4B, C**). PERMANOVA confirmed significant differences in overall community structure among the three groups (Bray-Curtis: R² = 0.223, P = 0.001; weighted UniFrac: R² = 0.257, P = 0.008), with significant separation between the DRS and OVX groups after Benjamini–Hochberg correction for pairwise tests (adjusted P = 0.012 and 0.010, respectively). At the phylum level (**Figure 4D**), *Firmicutes* and *Bacteroidota* were the predominant phyla in all groups (**Figure 4E**). At the genus level (**Figures 4F, G**), DRS treatment was associated with higher relative abundance of *Limosilactobacillus*, *Lactobacillus*, *Faecalibaculum*, *Odoribacter*, *Negativibacillus*, and *Enterorhabdus*, and a lower relative abundance of *Parasutterella* compared with the OVX group. The heatmap of genus-level abundance is presented in **Supplementary Figure 1H**. To further identify osteoporosis-associated bacterial biomarkers modulated by DRS, LEfSe identified several taxa differing among groups (**Supplementary Figure 2A**), including higher relative abundances of *Lactobacillus* and *Limosilactobacillus* in the DRS group. Although no individual taxon remained significant after Benjamini-Hochberg correction, the significant overall shift in community structure suggests that DRS modulates the gut microbiota in a distributed manner rather than through large changes in a few taxa. These taxa were therefore considered exploratory candidate biomarkers. To investigate the potential functional consequences of gut microbiota alterations, PICRUSt2 functional prediction analysis was performed (**Supplementary Figure 2C**), identifying amino acid metabolism and carbohydrate metabolism as the predominant predicted functional categories.

**Figure 4:**
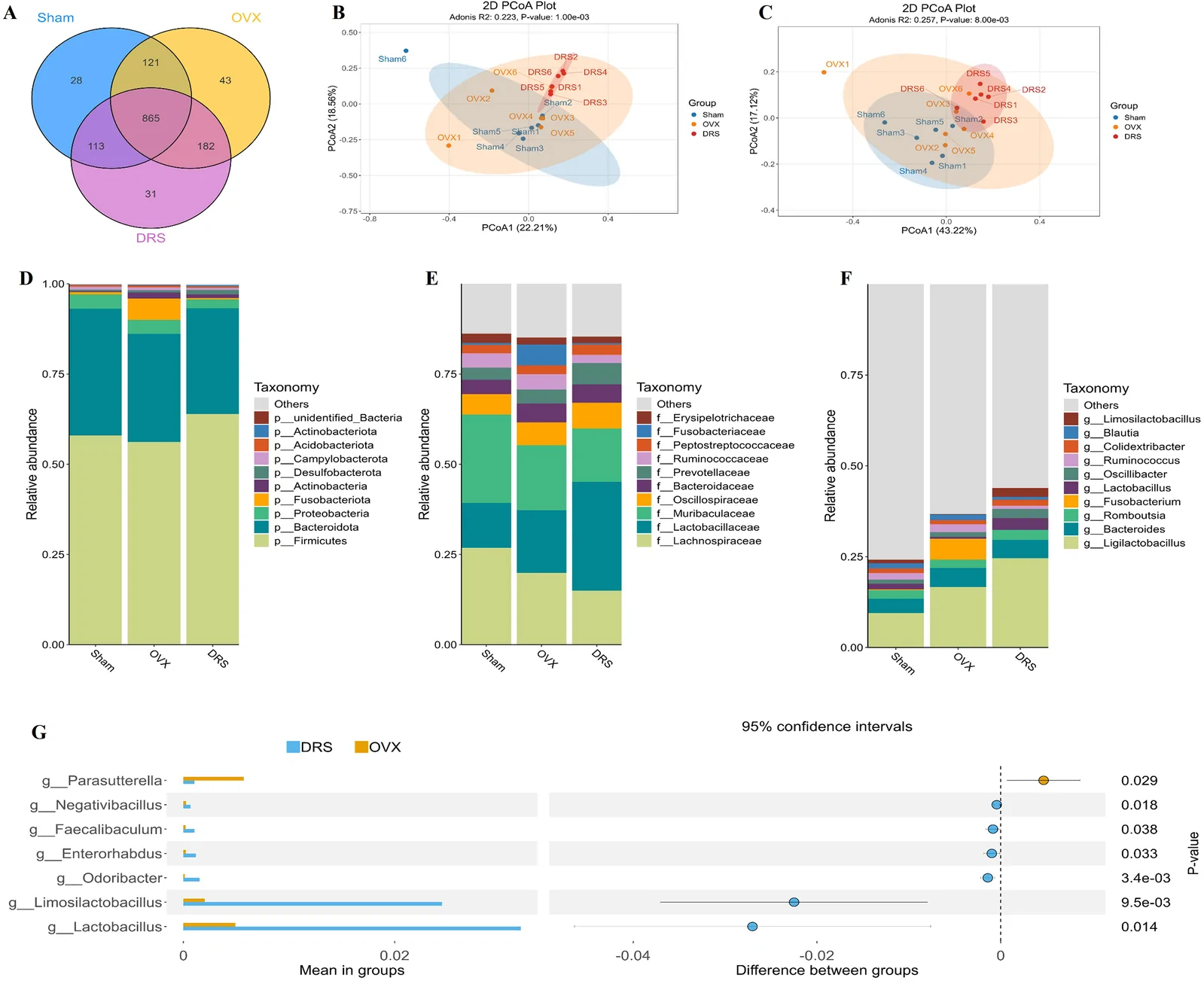
Effects of DRS on gut microbiota in OVX rats. **(A)** Venn diagram. **(B)** PCoA analysis based on Bray-Curtis and **(C)** weighted UniFrac distance. Relative abundance composition of gut microbiota at **(D)** phylum, **(E)** family, and **(F)** genus taxonomic levels across groups. **(G)** Genus-level taxa differing between the OVX and DRS groups (two-sided t-test, nominal P < 0.05; none remained significant after Benjamini-Hochberg correction). Bars show mean relative abundance; dots show between-group differences with 95% confidence intervals. Data are presented as mean ± SEM (n = 6).

### 3.5 DRS restores metabolic homeostasis in OVX rats

To investigate metabolic changes in osteoporosis, we performed metabolomic analysis. TICs of QC samples demonstrated consistent metabolite retention times and peak intensities with high overlap, confirming stable instrument performance and reliable data (**Supplementary Figures 3A, B**). PCA revealed distinct clustering within groups among Sham, OVX, and DRS groups (**Figure 5A**). Subsequently, orthogonal partial least squares-discriminant analysis score plots for Sham versus OVX and OVX versus DRS comparisons were constructed (**Supplementary Figures 3C, D**). Differential metabolites between Sham and OVX groups, as well as between OVX and DRS groups, were visualized using volcano plots (**Figures 5B, C**). Between Sham and OVX groups, 43 differential metabolites were identified (**Supplementary Table 1**), including 26 downregulated and 17 upregulated metabolites. Between OVX and DRS groups, 34 metabolites were differentially abundant (**Supplementary Table 2**), including 13 downregulated and 21 upregulated metabolites (**Figure 5D**). KEGG pathway enrichment analysis of differential metabolites indicated that metabolites altered by OVX were primarily enriched in tryptophan metabolism, taste transduction, purine metabolism, and phenylalanine metabolism (**Figure 5E**). In contrast, metabolites altered by DRS treatment were mainly enriched in purine metabolism, nucleotide metabolism, linoleic acid metabolism, fructose and mannose metabolism, and ABC transporters (**Figure 5F**).

**Figure 5:**
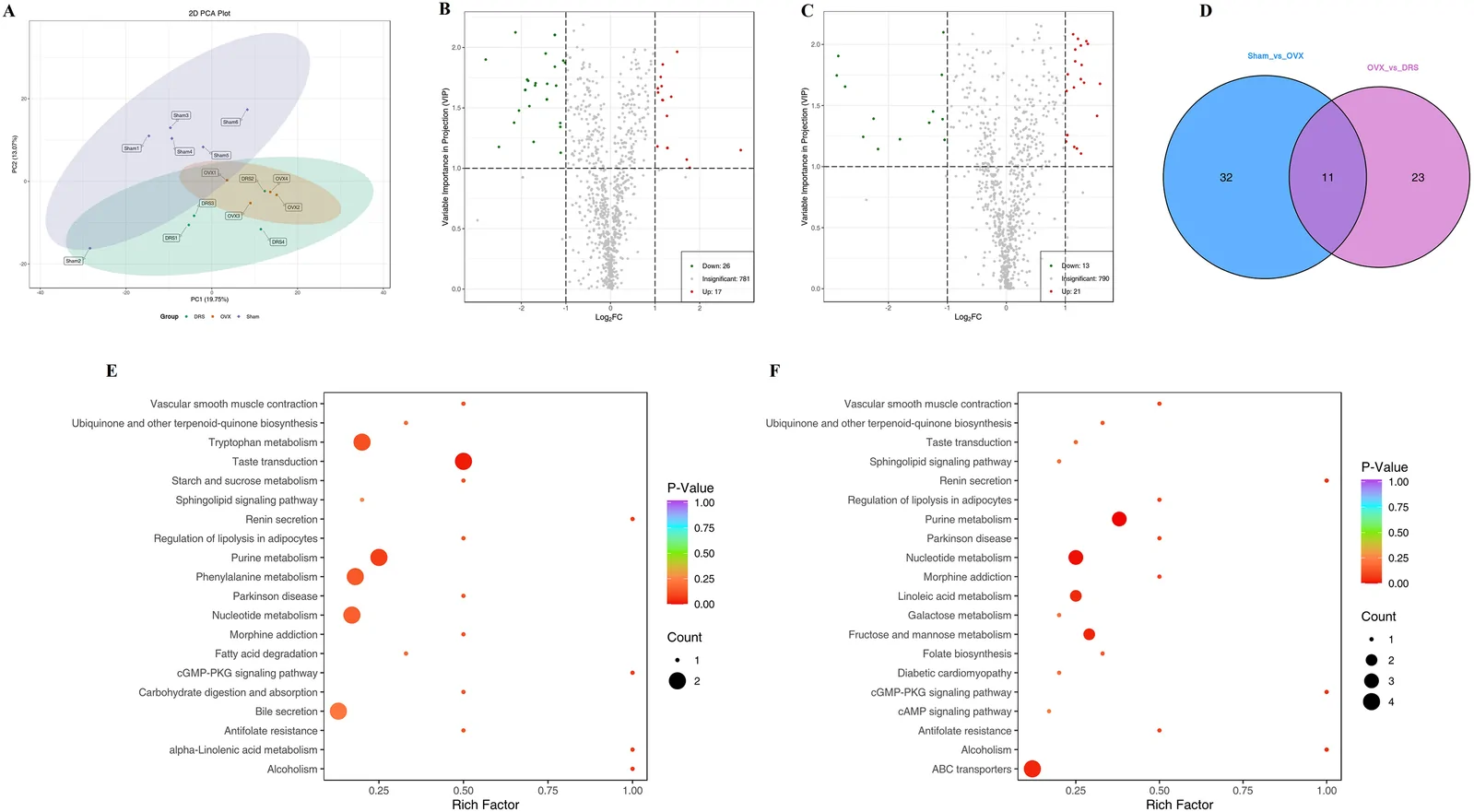
Effects of DRS on serum metabolites in OVX rats. **(A)** PCA plot. Volcano plots of differential metabolites in **(B)** Sham versus OVX and **(C)** OVX versus DRS. (D) Venn diagram. KEGG pathway enrichment of differential metabolites in **(E)** Sham versus OVX and **(F)** OVX versus DRS. In **(B, C)**, Up and Down indicate metabolites with higher or lower abundance in OVX relative to Sham **(B)** or in DRS relative to OVX **(C)**. n = 6 for the Sham group and n = 4 for the OVX and DRS groups.

### 3.6 Effect of DRS on the transcriptome in OVX rats

RNA sequencing analysis was performed on femoral tissues. PCA showed the overall distribution of transcriptomic profiles, with partial separation among the three groups (**Supplementary Figure 4B**). Genes with |log2 fold change| ≥ 1 and nominal P < 0.05 were used as an exploratory gene set for GO and KEGG overrepresentation analyses. This gene set included 629 genes in the Sham versus OVX comparison (493 upregulated and 136 downregulated in OVX rats **Figure 6A**; **Supplementary Table 3**) and 319 genes in the OVX versus DRS comparison (139 upregulated and 180 downregulated after DRS treatment **Figure 6B**; **Supplementary Table 4**). In addition, gene set enrichment analysis (GSEA) was performed on all genes ranked by log2 fold changes to detect coordinated changes at the gene-set level (**Supplementary Tables 5**-**8**).

GO-based GSEA showed positive enrichment of muscle-related gene sets in OVX rats, including sarcomere organization, myofibril assembly, contractile fiber formation, and striated muscle cell development (**Figure 6C**). In the OVX versus DRS comparison, these muscle-related gene sets showed negative enrichment, whereas collagen fibril organization and extracellular matrix organization showed positive enrichment (**Figure 6D**). A similar pattern was observed in the chord plot: *Col1a1* and *Col1a2* showed positive log2 fold changes after DRS treatment, whereas *Myh3*, *Myh8*, *Myog*, *Myf5*, and *Pax7* showed negative values (**Supplementary Figure 5B**). GO overrepresentation analysis of the exploratory gene set also showed enrichment of skeletal muscle development, extracellular matrix, and collagen-containing extracellular matrix terms (**Supplementary Figure 6A**). KEGG overrepresentation analysis showed enrichment of muscle contraction-related pathways in the Sham versus OVX comparison (**Supplementary Figure 6B**). Detailed GSEA results are provided in **Supplementary Tables 5**-**8**.

**Figure 6:**
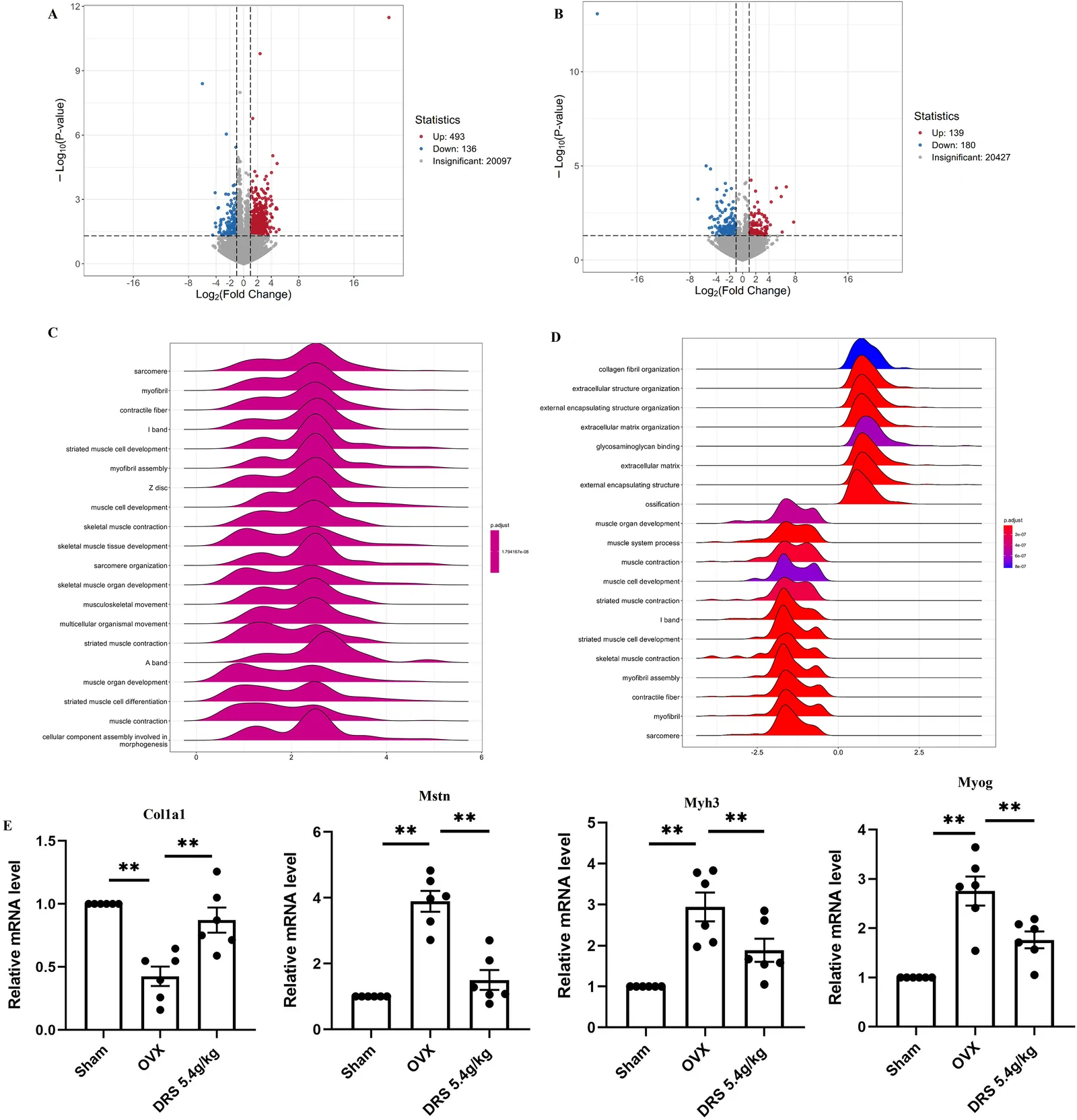
Effects of DRS on femoral transcriptomics in OVX rats. **(A, B)** Volcano plots of the exploratory gene sets in the Sham versus OVX and OVX versus DRS comparisons, respectively. Up and Down indicate higher or lower expression in OVX relative to Sham **(A)** or in DRS relative to OVX **(B)**. GSEA ridge plots of the GO database in **(C)** Sham versus OVX and **(D)** OVX versus DRS. **(E)** mRNA expression levels of selected candidate genes. Data in **(E)** are presented as mean ± SEM (n = 5). **P < 0.01.

RT-qPCR showed directional changes consistent with the RNA-seq results for the selected genes (**Figure 6E**). Compared with the Sham group, *Col1a1* expression was lower in the OVX group, whereas *Myog*, *Myh3*, and *Mstn* were higher. DRS treatment increased *Col1a1* expression and reduced the expression of *Myog*, *Myh3*, and *Mstn*.

### 3.7 DRS promotes osteoblast differentiation *in vitro*

To validate the pro-osteogenic effects of DRS observed *in vivo*, MC3T3-E1 cells were treated with serum collected from DRS-treated rats. Compared with the control group, DRS-containing serum dose-dependently enhanced ALP staining (**Figure 7A**), supporting its role in promoting osteoblast differentiation. Western blotting analysis further demonstrated that DRS-containing serum significantly upregulated BMP2 and the key osteogenic transcription factor RUNX2 (**Figures 7B–D**), suggesting potential activation of the BMP-2/Smad signaling pathway.

**Figure 7:**
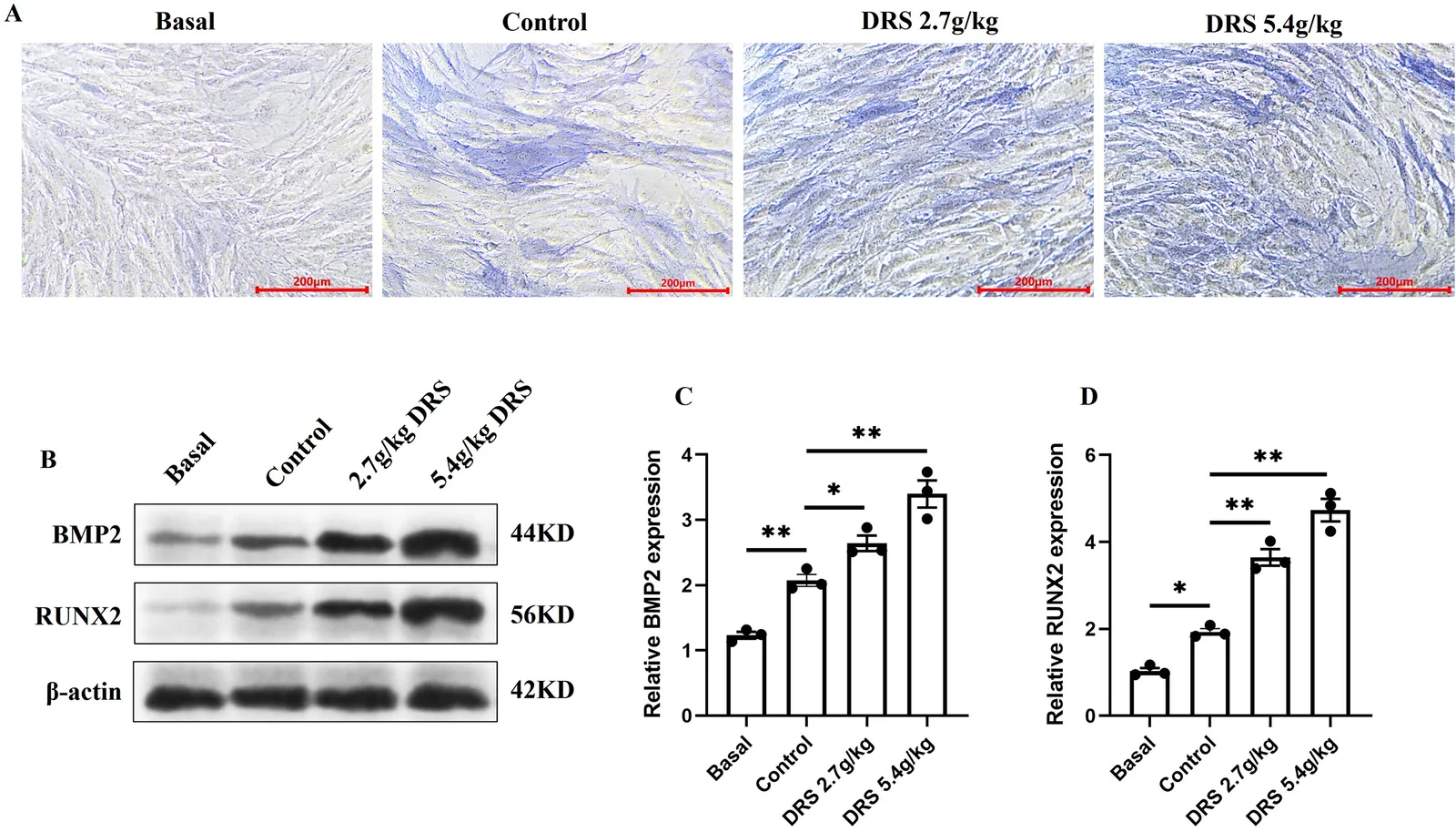
DRS-containing serum promotes osteoblast differentiation in MC3T3-E1 cells. **(A)** Representative images of ALP staining in MC3T3-E1 cells. **(B)** Representative Western blot images showing protein levels of BMP2, RUNX2. Quantitative analysis of BMP2 **(C)** and RUNX2 **(D)** protein expression normalized to b-actin. Data are presented as mean ± SEM (n = 3). *P < 0.05, **P < 0.01.

### 3.8 Integrated multi-omics analysis

Spearman correlation analysis was performed on the top 20 genus-level gut microbiota and on differentially expressed metabolites between OVX and DRS groups. DRS-enriched *Limosilactobacillus* and *Lactobacillus* exhibited significant positive correlations with the probiotic-associated metabolite (R)-2-hydroxy-3-phenylpropionic acid (23) and energy metabolism-related sugar alcohols (D-sorbitol, D-mannitol), while correlating negatively with oxidative stress markers (8-iso prostaglandin E2) (**Figure 8A**). Correlation analysis between bone parameters, bone metabolism factors, and key multi-omics molecules (genes, metabolites, and gut microbiota) revealed the following relationships (**Figure 8C**): collagen genes (*Col1a1*, *Col1a2*) correlated positively with E2 and negatively with LPS and bone turnover markers (ALP, CTX-1). Bone formation indices (BMD, BV/TV, Tb.N) showed consistent positive associations with both energy metabolites and probiotic abundance. LPS correlated positively with oxidative stress markers (8-iso prostaglandin E2, 15-keto prostaglandin E1) and purine metabolites (adenosine, inosine), and negatively with collagen genes (*Col1a1*, *Col1a2*) and D-sorbitol, indicating an association between gut barrier status and systemic inflammatory tone. The metabolome-transcriptome correlation map (**Figure 8B**) further showed that D-sorbitol and D-mannitol were negatively correlated with muscle-related genes (*Myog*, *Myh3*), while oxidative metabolites (15-keto prostaglandin E1, 8-iso prostaglandin E2) were positively correlated with these muscle-related genes. Integrated KEGG pathway analysis of metabolomic and transcriptomic data identified multiple pathways significantly enriched in the OVX and DRS groups. Among these, purine metabolism emerged as a candidate pathway potentially associated with the protective effects of DRS (**Figure 8D**).

**Figure 8:**
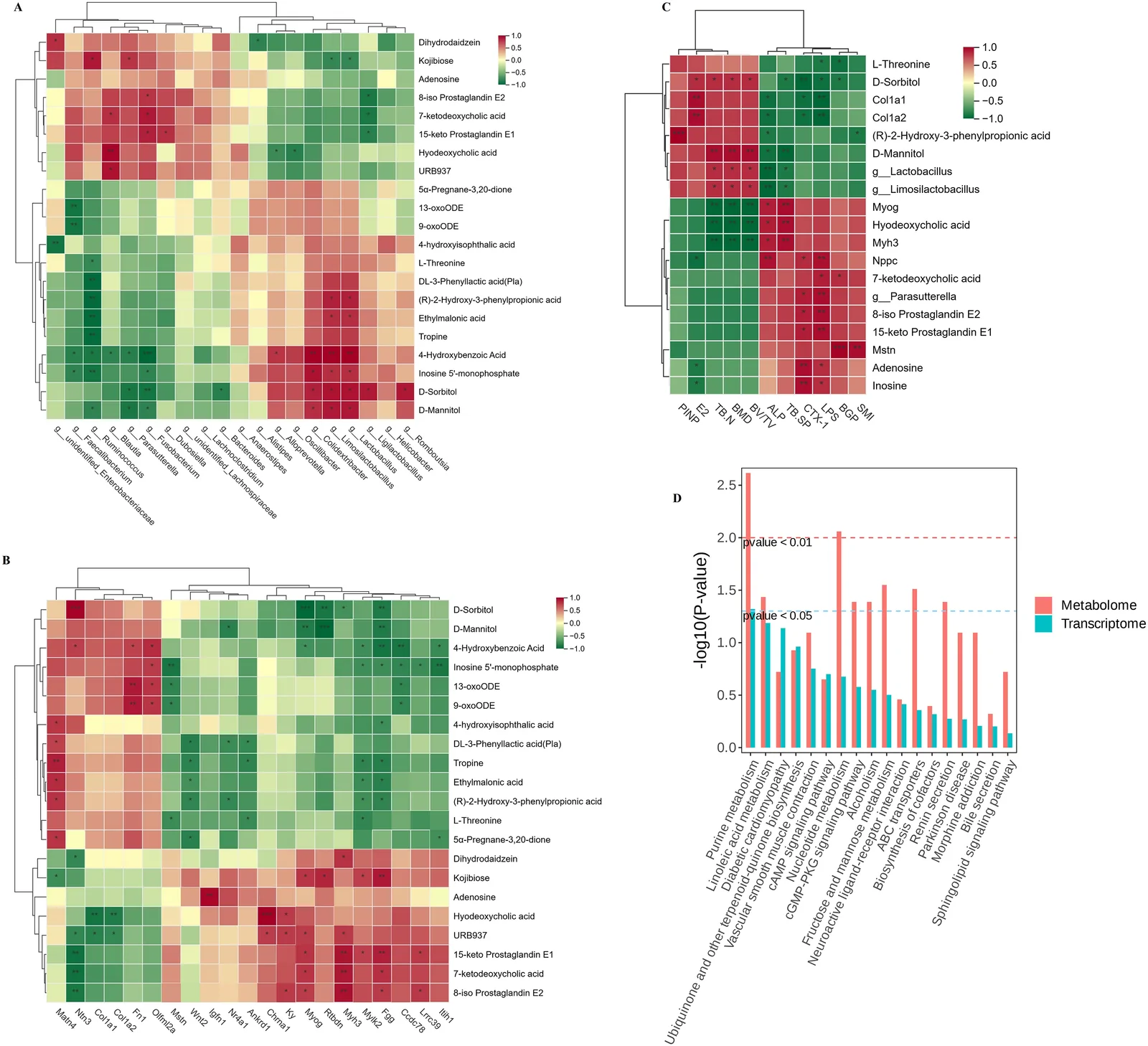
Multi-omics integrated analysis. **(A)** Spearman correlation analysis between genus-level microbiota and differential metabolites. **(B)** Pearson correlation analysis between metabolomics and transcriptomics. **(C)** Spearman correlation analysis between biochemical indicators and core omics molecules, and microbiota. **(D)** KEGG pathway enrichment bar plot of commonly enriched pathways between metabolomics and transcriptomics. Correlation analyses were performed on paired samples from the same animals [**(A, B)** n = 4 per group; **(C)** n = 3 per group] using nominal P values and were considered exploratory. *P < 0.05, **P < 0.01.

## 4 Discussion

This study investigated the protective effects and potential mechanisms of DRS in an OVX-induced rat model of osteoporosis by integrating analyses of the gut microbiome, metabolomics, and transcriptomics. The findings suggest that DRS may act through coordinated interactions along the gut-bone and bone-muscle axes, a pattern characteristic of multi-component herbal medicines.

In this study, DRS was associated with higher relative abundances of *Lactobacillus* and *Limosilactobacillus* and a lower abundance of *Parasutterella*. Multiple studies have demonstrated the bone-protective effects of *Lactobacillus* species and related genera in osteoporosis (24, 25). The abundance of these two genera correlated positively not only with bone formation indicators (BMD, BV/TV, and Tb.N), but also with serum levels of inosine 5’-monophosphate (IMP), D-sorbitol, and D-mannitol, while correlating negatively with inflammatory mediators such as 8-iso prostaglandin E2. Moreover, DRS markedly lowered serum LPS levels, a component of Gram-negative bacterial cell walls and a commonly used indicator of intestinal barrier integrity and systemic inflammatory load. The reduction in LPS aligns with the suppression of pro-inflammatory taxa such as *Parasutterella*. Collectively, these findings suggest that DRS may be associated with bone protection through both metabolic and inflammatory modulation by specific functional microbes, rather than simply increasing overall microbial diversity.

Metabolomic analysis revealed that DRS treatment significantly modulated purine metabolism. Specifically, DRS decreased adenosine and inosine and increased IMP, indicating that DRS treatment was associated with changes in purine-related metabolites. Previous studies have suggested that purine metabolic disturbance represents a prevalent pathological characteristic across different forms of osteoporosis, with its core detrimental effects including uric acid accumulation, excessive reactive oxygen species production, and chronic inflammation, thereby disrupting bone homeostasis (26). These metabolic changes provide insights into how DRS may affect the bone microenvironment. In addition, the significant positive correlation between IMP levels and the abundance of *Lactobacillus* and *Limosilactobacillus* further suggests an association between specific bacteria, purine metabolism, and bone stability.

Beyond metabolic integration, bulk RNA-seq of femoral tissue showed increased expression of muscle-related genes (*Myog*, *Myh3*, *Mstn*) together with downregulation of osteogenic genes (*Col1a1*, *Col1a2*) in OVX rats. These findings suggest that estrogen deficiency may be associated with an altered femoral transcriptomic signature; however, further validation using techniques such as cell-marker-guided RNA *in situ* hybridization or single-cell RNA sequencing is needed to confirm the cellular origin of these transcripts. DRS intervention partially reversed these changes and increased osteogenic marker expression. The multi-omics integration further showed that energy-related sugar alcohols (D-sorbitol, D-mannitol) correlated negatively with *Myog* and *Myh3* but positively with bone formation indices (BMD, BV/TV), whereas the oxidative stress marker 8-iso prostaglandin E2 correlated positively with muscle-related genes. This pattern does not prove causation on its own, but it offers system-level clues about how muscle and bone metabolism may be coupled. *In vitro*, treatment with DRS-containing serum enhanced osteoblast differentiation and upregulated BMP2 and RUNX2 expression. Together with our previous finding that DRS suppresses osteoclastogenesis in RAW264.7 cells (18), these results suggest that DRS is associated with bone-protective effects involving modulation of both osteoblast and osteoclast activity, potentially through effects on bone cells as well as systemic changes associated with muscle-bone crosstalk.

Several limitations should be noted. First, the associations among gut microbiota composition, purine metabolism, and bone phenotypes derive from parallel omics profiling and correlation analyses and should therefore be regarded as hypothesis-generating. Further intervention studies, including fecal microbiota transplantation, antibiotic-mediated microbiota depletion, and targeted metabolite supplementation, will be necessary to validate the proposed relationships. Second, the DRS-containing serum experiment cannot distinguish direct effects of DRS-derived constituents or metabolites from indirect effects mediated by systemic changes in donor animals.

## 5 Conclusion

In conclusion, this study characterized the multi-level regulatory effects of DRS against OVX-induced osteoporosis through integrated phenotypic, microbial, metabolic, and transcriptomic analyses. Our findings show that DRS was associated with restoration of beneficial gut bacteria, modulation of purine and energy metabolism, lowering of systemic inflammatory burden, and a tendency toward normalization of the expression of osteogenic and muscle-related genes in femoral tissue. Overall, this work provides a multi-omics view of the system-level mechanisms of complex herbal medicines and suggests potential applications of ethnic medicines in treating osteosarcopenia.

## Data Availability

The datasets presented in this study can be found in online repositories. The names of the repository/repositories and accession number(s) can be found below:https://www.ncbi.nlm.nih.gov/, PRJNA1413957https://www.ncbi.nlm.nih.gov/, PRJNA1398650https://ngdc.cncb.ac.cn/bioproject/browse/PRJCA057568, PRJCA057568https://ngdc.cncb.ac.cn/bioproject/browse/PRJCA057568, PRJCA057568.

## Glossary

ALP: Bone-specific alkaline phosphatase
ASVs: Amplicon sequence variants
BGP: Osteocalcin
BMD: Bone mineral density
BV/TV: Bone volume fraction
CMC-Na: Sodium carboxymethyl cellulose
CTX-1: C-telopeptide of type I collagen
DRS: Huangtcia renifolia ethanol extract
E2: Estradiol
ESI: Electrospray Ionization
FPKM: fragments per kilobase of transcript per million fragments mapped
GO: Gene Ontology
GSEA: Gene Set Enrichment Analysis
H&E: Hematoxylin-eosin
KEGG: Kyoto Encyclopedia of Genes and Genomes
LC-MS/MS: Liquid chromatography-tandem mass spectrometry
LPS: Lipopolysaccharide
LDA: Linear discriminant analysis
MRM: Multiple reaction monitoring
OPG: Osteoprotegerin
OPLS-DA: Orthogonal partial least squares-discriminant analysis
OVX: Ovariectomized
PCA: Principal component analysis
PCoA: Principal coordinates analysis
PINP: Procollagen type I N-terminal propeptide
qPCR: Quantitative polymerase chain reaction
RANK: Receptor activator of nuclear factor κB
RANKL: Receptor activator of nuclear factor κB ligand
RT-qPCR: Real-time quantitative PCR
SMI: Structural model index
Tb.N: Trabecular number
Tb.Sp: Trabecular separation
Tb.Th: Trabecular thickness
UPLC-MS/MS: Ultra-performance liquid chromatography-tandem mass spectrometry
