# Supplementary material for: Multi-omics analysis explores the potential mechanisms of Huangtcia renifolia ethanolic extract against osteoporosis in ovariectomized rats
Source: Front Endocrinol (Lausanne). 2026 Sep 30;17:1795581. doi: 10.3389/fendo.2026.1795581 (PMC13663029; doi:10.3389/fendo.2026.1795581)
Supplement: Supplementary file 1 [file DataSheet1.docx]

**Supplementary Figure 1.** Effects of DRS on gut microbiota diversity and composition in OVX rats. (A) Rarefaction curves of ASV numbers against sequencing depth in Sham, OVX, and DRS groups. Each curve represents an individual sample. (B-G) Alpha diversity indices including (B) Chao1, (C) Ace, (D) observed ASVs, (E) Good's Coverage, (F) Shannon, and (G) Simpson indices among Sham, OVX, and DRS groups. (H) Heatmap showing the relative abundance of genus-level microbiota across groups.

**Supplementary Figure 2.** Comparative analysis of gut microbiota community differences among three groups. (A) Linear Discriminant Analysis (LDA) effect size bar chart of taxa identified by LEfSe; (B) Cladogram illustrating the phylogenetic relationships of differential species; (C) PICRUSt2-predicted KEGG pathway abundance diagram.

**Supplementary Figure 3.** Metabolic profiling analysis of DRS treatment in OVX rats. Total ion chromatogram (TIC) overlay of QC samples analyzed using (A) Amide column and (B) T3 column. OPLS-DA scores plot showing metabolic profile differences between (C) Sham and OVX groups and (D) OVX and DRS groups. Hierarchical clustering heatmap of differential metabolites between (E) Sham and OVX groups and (F) OVX and DRS groups.

**Supplementary Figure 4.** Gene expression profiling analysis of DRS treatment in OVX rats. (A) Box plot of log10(FPKM) values across all samples. (B) PCA plot based on FPKM values. Hierarchical clustering heatmaps of the exploratory gene sets from the (C) Sham versus OVX and (D) OVX versus DRS comparisons.

**Supplementary Figure 5.** Functional enrichment analysis of differential genes. (A) GO enrichment chord diagram showing functional categories between Sham and OVX groups. (B) GO enrichment chord diagram showing functional categories between OVX and DRS groups. (C) KEGG pathway enrichment chord diagram between Sham and OVX groups. (D) KEGG pathway enrichment chord diagram between OVX and DRS groups.

**Supplementary Figure 6.** Exploratory GO and KEGG overrepresentation analyses of femoral transcriptomes. (A) GO and (B) KEGG enrichment dot plots for the exploratory gene sets from the indicated pairwise comparisons. Dot size indicates the number of associated genes, and color indicates the q value.
